# Evaluation of the use of unipolar voltage amplitudes for detection of myocardial scar assessed by cardiac magnetic resonance imaging in heart failure patients

**DOI:** 10.1371/journal.pone.0180637

**Published:** 2017-07-05

**Authors:** Uyên Châu Nguyên, Francesco Maffessanti, Masih Mafi-Rad, Giulio Conte, Stef Zeemering, François Regoli, Maria Luce Caputo, Antonius M. W. van Stipdonk, Sebastiaan C. A. M. Bekkers, Daniel Suerder, Tiziano Moccetti, Rolf Krause, Frits W. Prinzen, Kevin Vernooy, Angelo Auricchio

**Affiliations:** 1 Department of Physiology, Cardiovascular Research Institute Maastricht, Maastricht University, Maastricht, the Netherlands; 2 Department of Cardiology, Maastricht University Medical Center, Maastricht, the Netherlands; 3 Center for Computational Medicine in Cardiology, Institute of Computational Science, Università della Svizzera italiana, Lugano, Switzerland; 4 Department of Cardiology, Fondazione Cardiocentro Ticino, Lugano, Switzerland; Scuola Superiore Sant'Anna, ITALY

## Abstract

**Background:**

Validation of voltage-based scar delineation has been limited to small populations using mainly endocardial measurements. The aim of this study is to compare unipolar voltage amplitudes (UnipV) with scar on delayed enhancement cardiac magnetic resonance imaging (DE-CMR).

**Methods:**

Heart failure patients who underwent DE-CMR and electro-anatomic mapping were included. Thirty-three endocardial mapped patients and 27 epicardial mapped patients were investigated. UnipV were computed peak-to-peak. Electrograms were matched with scar extent of the corresponding DE-CMR segment using a 16-segment/slice model. Non-scar was defined as 0% scar, while scar was defined as 1–100% scar extent.

**Results:**

UnipVs were moderately lower in scar than in non-scar (endocardial 7.1 [4.6–10.6] vs. 10.3 [7.4–14.2] mV; epicardial 6.7 [3.6–10.5] vs. 7.8 [4.2–12.3] mV; both *p*<0.001). The correlation between UnipV and scar extent was moderate for endocardial (R = -0.33, *p*<0.001), and poor for epicardial measurements (R = -0.07, *p*<0.001). Endocardial UnipV predicted segments with >25%, >50% and >75% scar extent with AUCs of 0.72, 0.73 and 0.76, respectively, while epicardial UnipV were poor scar predictors, independent of scar burden (AUC = 0.47–0.56). UnipV in non-scar varied widely between patients (*p*<0.001) and were lower in scar compared to non-scar in only 9/22 (41%) endocardial mapped patients and 4/19 (21%) epicardial mapped patients with scar.

**Conclusion:**

UnipV are slightly lower in scar compared to non-scar. However, significant UnipV differences between and within patients and large overlap between non-scar and scar limits the reliability of accurate scar assessment, especially in epicardial measurements and in segments with less than 75% scar extent.

## Introduction

The measurement of voltage amplitudes to distinguish scar tissue from viable myocardium is widely employed in invasive cardiology and electrophysiology including ablation therapy, targeted delivery of biological therapy, and cardiac resynchronization therapy (CRT).[1–5] Low voltage amplitudes are considered to be associated with scar, while high voltage amplitudes are regarded as viable myocardium.[2]

Delayed enhancement cardiac magnetic resonance (DE-CMR) is currently the gold standard for myocardial scar delineation. Several studies have correlated low voltage amplitudes and DE-CMR defined scar, from which most have been conducted in porcine models with modestly reduced left ventricular (LV) ejection and a well-demarcated scar zone.[2, 6–8] However, characteristics of these models do not represent the scar heterogeneity noticed in heart failure (HF) patients, limiting the generalizability of the results. Additionally, validation studies of voltage-based scar delineation conducted in ventricular tachycardia (VT) or HF patients have been limited by the small numbers of patients investigated, was particularly focused on endocardial measurements, and do not provide data on the effectiveness at the individual level.[3, 9, 10]

The aim of the present study was to compare voltage amplitude measurements with scar on DE-CMR in ischemic and non-ischemic HF patients. In order to increase the generalizability of the study results, this investigation was performed in a cohort with endocardial measurements and a cohort with epicardial measurements. To this purpose, we (1) compared voltage amplitudes with the presence of DE-CMR defined scar, (2) correlated voltage amplitudes with segmental scar extent, and (3) evaluated the diagnostic accuracy of voltage amplitudes in detecting scar.

## Methods

### Study population

**Fig 1:**
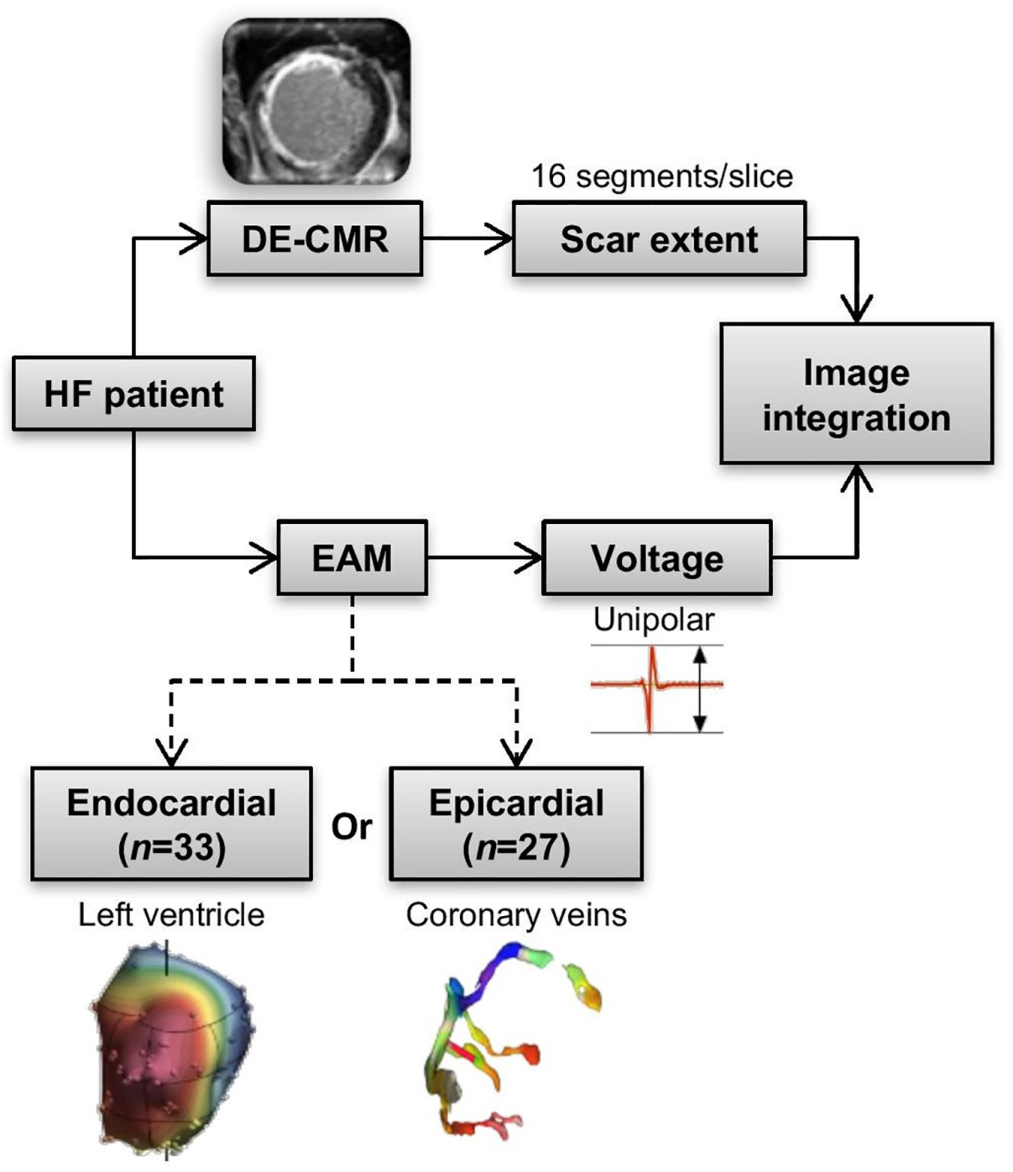
Study workflow. DE-CMR = delayed enhancement cardiac magnetic resonance imaging, EAM = electro-anatomic mapping, HF = heart failure.

HF patients (New York Heart Association class, NYHA ≥I) who underwent DE-CMR and epicardial (coronary venous) electro-anatomic mapping at Maastricht University Medical Center or endocardial electro-anatomic mapping (EAM) at Cardiocentro Ticino for biological therapy or CRT were retrospectively included. The workflow of the study is illustrated in Fig 1. The institutional review board from Maastricht University Medical Center and the Ethics Committee of Canton Ticino approved the study protocol and waived the need for informed consent.

### Cardiac magnetic resonance imaging

All CMR scans were part of standard care. Acquisition protocols and post-processing data have been extensively described previously.[11–13] In brief, CMR images were acquired with a 3-Tesla scanner (Siemens Magnetom Skyra, Germany) in CCT or a 1.5–3.0-Tesla scanner (Philips Intera/Ingenia/Achieva, Best, the Netherlands) in MUMC. ECG-gated cine images were obtained to determine LV functions. DE-CMR images were acquired with a phase sensitive inversion recovery sequence for CCT (typical voxel size 1.2×1.2×8 mm) and a 2D inversion gradient echo sequence for MUMC (typical voxel size 0.6x0.6x0.8 mm) 7–15 minutes after an intravenous bolus (0.2 mmol/kg for CCT and 0.15 mmol/kg for MUMC) of Gadobutrol (Gadovist Bayer Schering Pharma, Zurich, Switzerland). Endocardium and epicardium were manually traced from short-axis DE-CMR images using software programmed in MATLAB R2015b (MathWorks, Natick, MA) for CCT and CAAS MRV 3.4 (Pie Medical Imaging, Maastricht) for MUMC. Scar was semi-automatically quantified with the full-width-half-maximum criterion. All segmentations were performed by an investigator supervised by an experienced institutional CMR reader with over 10 years of experience (EACVI level3). Each DE-CMR short-axis slice was subdivided in 16-segments for which the scar extent was computed as the quantified scar area divided by the total segmental area. Non-scar was defined as 0% scar extent, while “any-scar” was defined as 1–100% scar extent. Any-scar was further subdivided in groups according to 1–25%, 26–50%, 51–75%, and 76–100% scar extent.

### Endocardial electro-anatomic mapping and DE-CMR integration

Endocardial EAM was performed using the NOGA ® XP Cardiac Navigation System (Biosense Webster, Johnson & Johnson Company) in CCT as previously described.[11] In brief, unipolar electrograms and catheter tip trajectories in 3D space were simultaneously recorded at the entire LV endocardium (filter settings, bandpass: 1–240 Hz). The NOGA ® XP system automatically discards points with insufficient wall contact.

All the acquired signals were temporally aligned using the simultaneously recorded surface ECG. Endocardial EAMs were integrated with DE-CMR offline by a rigid registration algorithm using a two-step algorithm developed in MATLAB. First, the cloud of NOGA points was translated to make their center of gravity overlapping with the center of gravity of the endocardial cavity segmented from the cine CMR images at end-diastole. Second, an iterative closest point approach was used to minimize the Euclidean distance between the NOGA points and the endocardial contours from DE-CMR.

### Epicardial electro-anatomic mapping and DE-CMR integration

Epicardial electrograms were derived from coronary venous EAMs in the MUMC, using EnSite NavX as described previously.[12] Briefly, a guidewire permitting unipolar sensing and pacing was inserted into the coronary sinus and manipulated to all tributaries, creating an anatomic 3D map of the coronary veins while simultaneously recording electrograms (filter settings, bandpass: 2–300 Hz) and a surface ECG during intrinsic rhythm. Electrograms with poor signal indicating poor contact were manually discarded during the procedure. Epicardial EAMs were integrated with DE-CMR using EnSite NavX by setting anatomical landmarks at both the EAM and DE-CMR geometry. The EAM coordinate system was subsequently superimposed and adjusted to the DE-CMR geometry using a dynamic registration algorithm, allowing local refinement while leaving other areas unaffected.

### Electrogram analyses

Endocardial and epicardial peak-to-peak unipolar voltage amplitudes (UnipV) were automatically computed using custom software programmed in MATLAB. Electrogram locations were matched with scar extent of the corresponding DE-CMR segment.

### Statistical analysis

Statistical analyses for the baseline characteristics were performed using SPSS 24.0 software (SPSS inc. Chicago, Illinois). Continuous variables are expressed as mean±SD or median with IQR and categorical variables in total numbers and frequencies. Two-tailed analyses were used and a *p*<0.05 was considered significant. The correlation between UnipV and DE-CMR scar extent was evaluated by partial correlation analysis controlled for inter-patient differences. UnipV from different scar groups were compared with non-scar using ANOVA tests with post-hoc Bonferroni correction. UnipV of any-scar vs. non-scar was compared using linear mixed models with patients as random effect and DE-CMR scar as fixed effect. Any-scar was compared with non-scar in individual patients using Mann-Whitney U tests. Kruskal Wallis tests were used to compare UnipV from non-scar and any-scar between individual patients. ROC curves were generated to determine the diagnostic accuracy of UnipV amplitudes in detecting segments with any-scar (1–100%) and segments with several severities of scar: >25%, >50%, and >75% scar.

## Results

### Study characteristics

A total of 60 HF patients were investigated: a cohort of 33 patients underwent extensive endocardial mapping (Group 1), and another cohort of 27 patients underwent epicardial mapping (Group 2). None of the patients had mapping at both endocardial or epicardial site. Clinical characteristics were typical of patients selected for CRT or biological therapy. Patients’ baseline characteristics are summarized in Table 1.

**Table 1:** Patient characteristics.

		Endocardial mapping (Group 1)	Epicardial mapping (Group 2)
Demographics			
	Patient number	33	27
	Age (years)	69.2±11.1	68.5±9.5
	Male	24(73)	20(74)
	BMI (kg/m2)	28.2±3.6	27.2±4.2
	Ischemic cardiomyopathy	19(58)	19(70)
	Non-ischemic cardiomyopathy	14(42)	8(30)
	NYHA (I/II/III)	2(6)/14(42)/17(52)	0(0)/18(67)/9(33)
	Biological therapy	8 (24%)	0 (0%)
	CRT	25 (76%)	27 (100%)
CMR LV function			
	LV mass (g)	159±39	156±46
	Scar present	22(67)	19(70)
	Scar (% LV mass)	19±20	11±11
	EF (%)	27±8	24±7
	EDV (ml)	270±89	291±87
	ESV (ml)	199±78	223±74
ECG characteristics			
	Sinus rhythm	30(91)	26(96)
	Atrial fibrillation	3(9)	1(4)
	QRS duration (ms)	153±27	153±22
	LBBB	21(62)	20(74)
	IVCD	5(15)	7(26)
Medication			
	Antiplatelet	28(82)	15(56)
	Coumarins	11(32)	19(70)
	Beta-blockers	33(97)	26(96)
	Calcium antagonists	2(6)	4(15)
	Ace-inhibitor/ARB	30(88)	24(89)
	Nitrates	4(12)	18(67)
	Diuretics	26(77)	21(78)
	Statin	20(59)	19(70)

Values are mean±SD or *n*(%). ACE = angiotensin-converting-enzyme, ARB = angiotensin-II-receptor-blocker, BMI = body-mass-index, CMR = cardiac magnetic resonance, CRT = cardiac resynchronization therapy, EDV = end-diastolic volume, ESV = end-systolic volume, IVCD = interventricular conduction disturbance, LBBB = left bundle branch block, LV = left ventricular, LVEF = LV ejection fraction, NYHA = New York Heart Association.

### Electro-anatomic mapping and DE-CMR image integration

A mean of 200±56 endocardial mapping points per patient were acquired in Group 1 for a total of 6612 endocardial electrograms, whereas a mean of 69±26 epicardial mapping points were acquired in Group 2 for a total of 1871 epicardial electrograms. EAMs were integrated with DE-CMR with a Euclidean distance of 6.9±4.8 mm for endocardial and 4.7±1.1 mm for epicardial measurements.[12] After excluding electrograms from the base, LV outflow tract, and right ventricle, 6543 endocardial electrograms were analyzed from non-scar (*n* = 3002) and any-scar (*n* = 3541) and 1330 epicardial electrograms from non-scar (*n* = 778) and any-scar (*n* = 552).

### Voltage amplitudes in scar vs. non-scar

**Fig 2:**
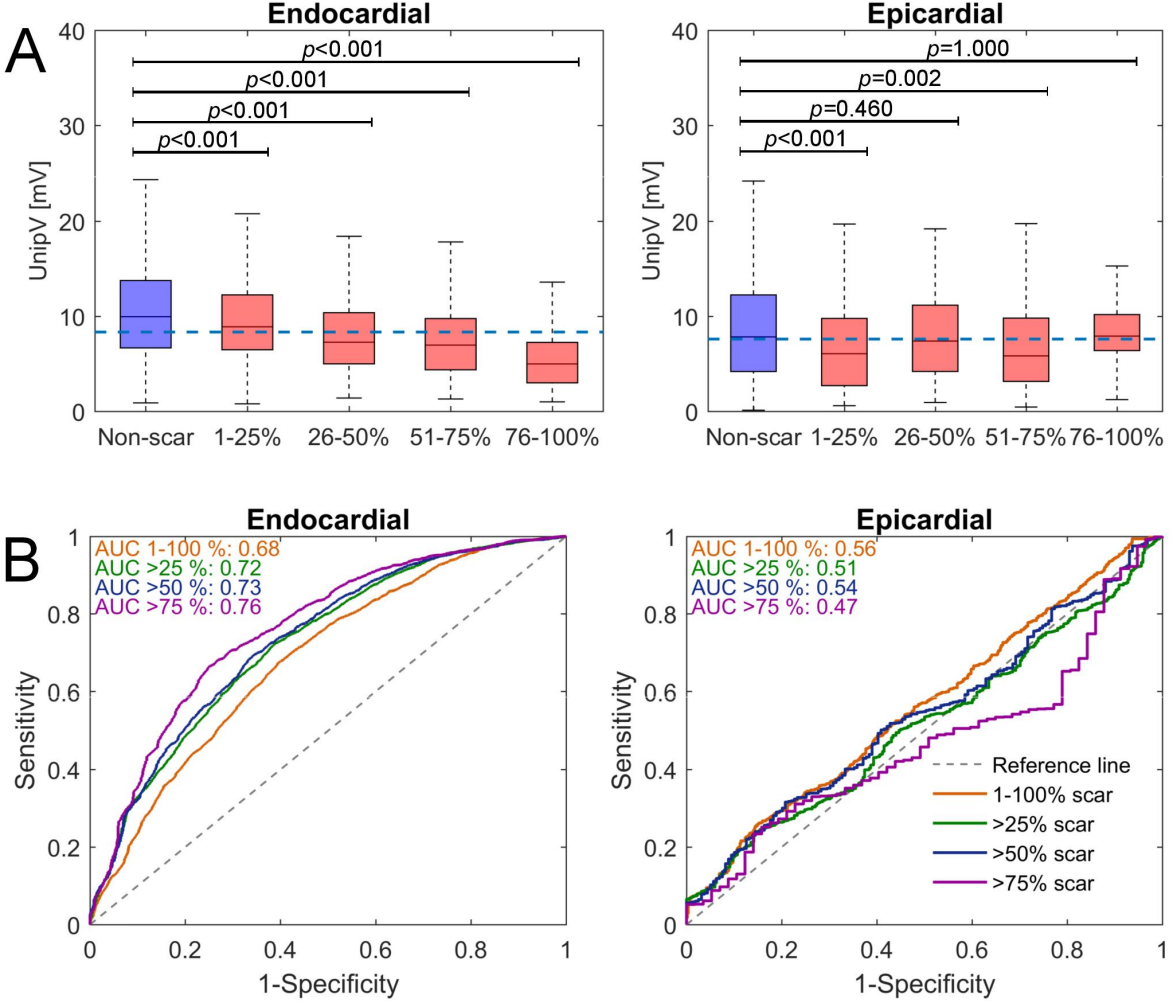
A. Unipolar voltage (UnipV) distribution grouped according to DE-CMR defined scar extent (red) and compared with non-scar (blue). Dashed blue lines represent optimal thresholds: 8.3 mV for the endocardial measurements, and 7.6 mV for the epicardial measurements. The tops and bottoms of each "box" represent the 25th and 75th percentiles of the subgroups, respectively. Distances between the tops and bottoms of each box represent the interquartile ranges. The horizontal middle line in the box represents the median value. The whiskers (vertical lines) above and below each box are drawn from the ends of the interquartile ranges to the furthest observations within the whisker length. B. ROC curves for UnipV in identifying DE-CMR defined segments with any-scar (1–100% scar) and segments with several severities of scar (>25%, >50%, and >75%). Note that the endocardial measurements are superior in detecting scar, independent of scar burden, although only segments with >75% scar can be properly detected (AUC 0.76) using endocardial measurements.

Endocardial and epicardial UnipV were slightly lower in segments with any-scar compared to non-scar (Table 2). However, variation in each category was large and box-plots in Fig 2A illustrate high overlap between UnipV within both non-scar and any-scar. The overlap was larger for epicardial than endocardial measurements, and particularly present in segments with <75% scar. Median UnipV gradually decreased with increasing scar extent for endocardial UnipV, but not for epicardial UnipV.

**Table 2:** Unipolar voltage amplitude distribution in non-scar and scar.

Substrate	Endocardial (Group 1)		*p*-value	Epicardial (Group 2)		*p*-value
	Electrogram (*n*)	Median (IQR)		Electrogram (*n*)	Median (IQR)	
Non-scar						
0%	3002	10.3 (7.4–14.2)	-	778	7.8 (4.2–12.3)	-
Any-scar^a^						
1–100%	3541	7.1 (4.6–10.6)	<0.001	552	6.7 (3.6–10.5)	<0.001
Scar groups^b^						
1–25%	1535	8.6 (6.0–12.3)	<0.001	234	6.1 (2.7–9.8)	<0.001
26–50%	675	7.0 (4.7–10.4)	<0.001	159	7.4 (4.2–11.2)	0.442
51–75%	501	7.0 (4.3–9.8)	<0.001	102	5.8 (3.2–9.9)	0.002
76–100%	830	5.0 (3.0–7.3)	<0.001	57	7.9 (6.4–10.3)	1.000

^*a*^*P*-values between non-scar and any-scar are based on linear mixed models.

^b^*P*-values for the scar-groups are based on ANOVA tests with post-hoc Bonferroni correction between non-scar and different scar-groups (0% vs. 1–25% scar, 0% vs. 26–50% scar etc.). UnipV are displayed as median and IQR.

**Fig 3:**
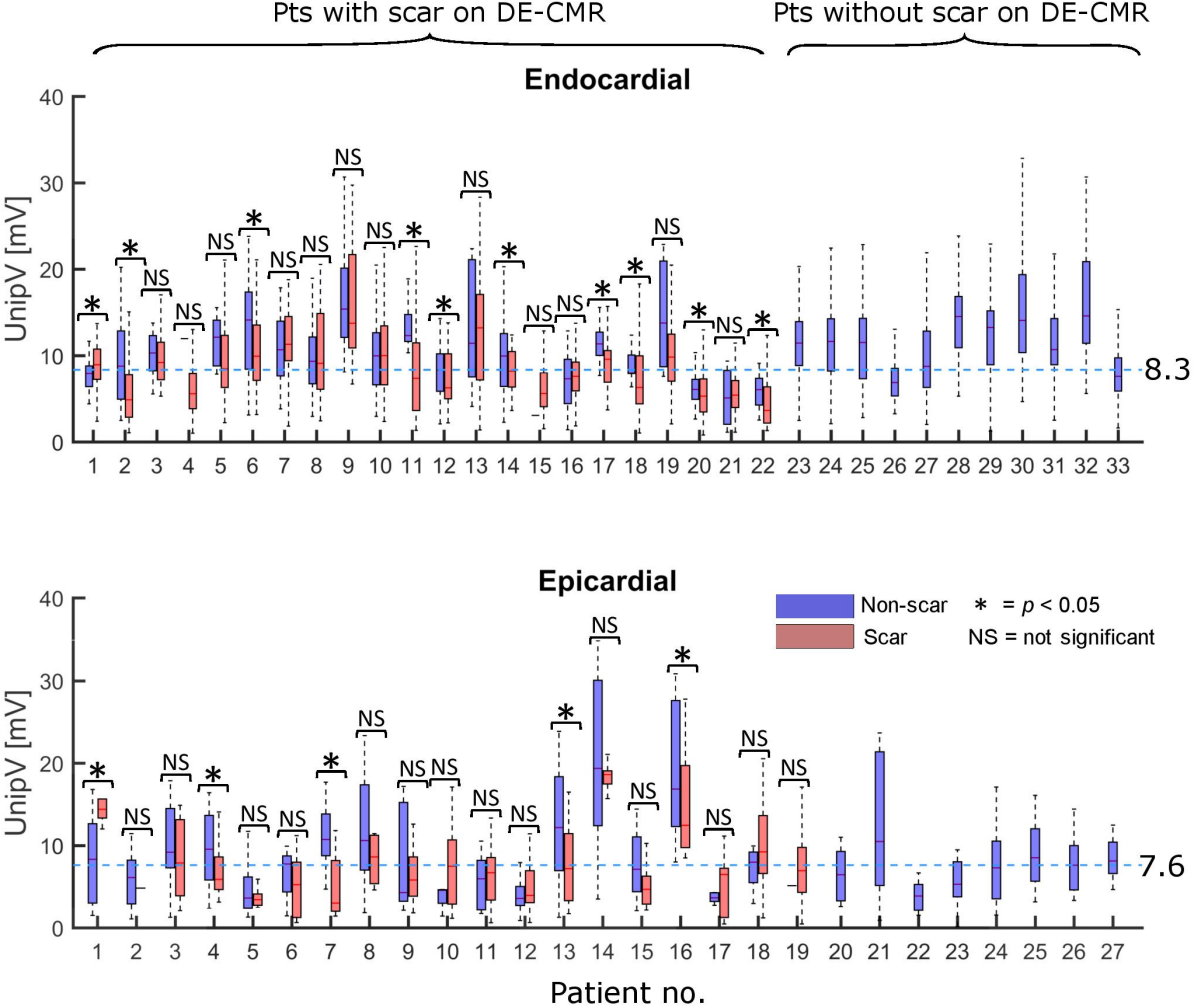
Endocardial and epicardial box-plots of the unipolar voltage (UnipV) distribution per individual patient in non-scar (0%, blue) and any-scar (1–100%, red). P-values per individual patient are based on Mann-Whitney U tests; a *p*-value of ≤0.05 is considered as significant (*), while p-values >0.05 are regarded as not significant (NS). Note that significantly lower UnipV in scar compared to non-scar were only present in the minority of patients. Patients without scar on delayed enhancement cardiac magnetic resonance imaging (DE-CMR) are plotted on the right. Dashed blue lines represent optimal voltage thresholds. Note the large variation in voltages from non-scar between individual patients.

When comparing non-scar UnipV distributions (blue boxplots in Fig 3) between individual patients, large variations in endocardial (median range: 5.1–15.4 mV, *p*<0.001) and epicardial (median range: 3.6–19.4 mV, *p*<0.001) UnipV between patients were observed, indicating that non-scar UnipV varied greatly between individuals. As demonstrated in Fig 3, UnipV in any-scar (red boxplots) were only significantly lower than non-scar (blue boxplots) in 9/22 (41%) endocardial mapped patients and 4/19 (21%) epicardial mapped patients.

### Correlation of voltage amplitudes with scar extent

**Fig 4:**
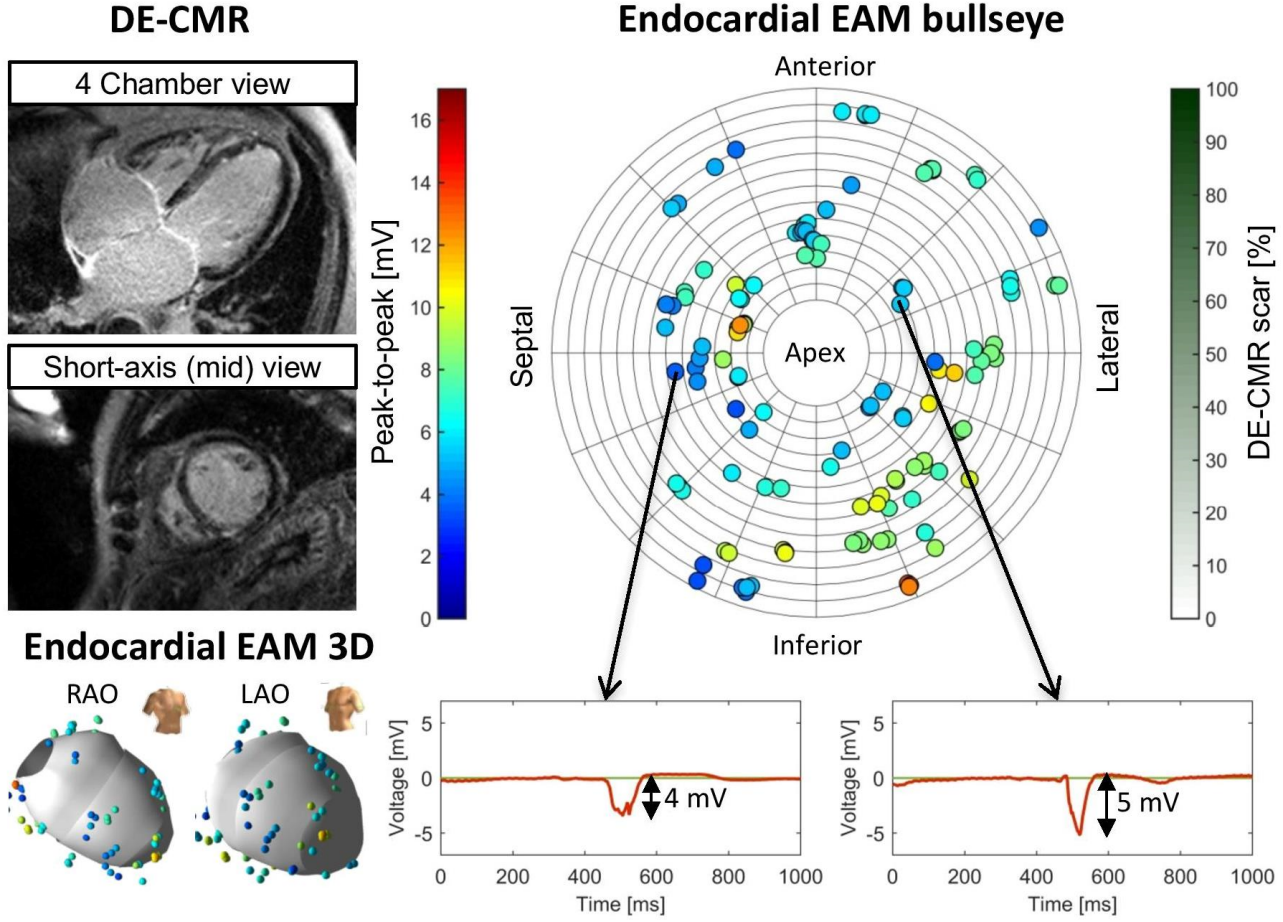
Representative patient (endocardial no.26) from without scar on delayed enhancement cardiac magnetic resonance imaging (DE-CMR). All unipolar electrograms were in non-scar, but still low unipolar voltage amplitudes (UnipV) were measured, demonstrating a lack of correlation between normal UnipV and non-scar. EAM = electro-anatomic mapping, LAO = left anterior oblique, RAO = right anterior oblique.

**Fig 5:**
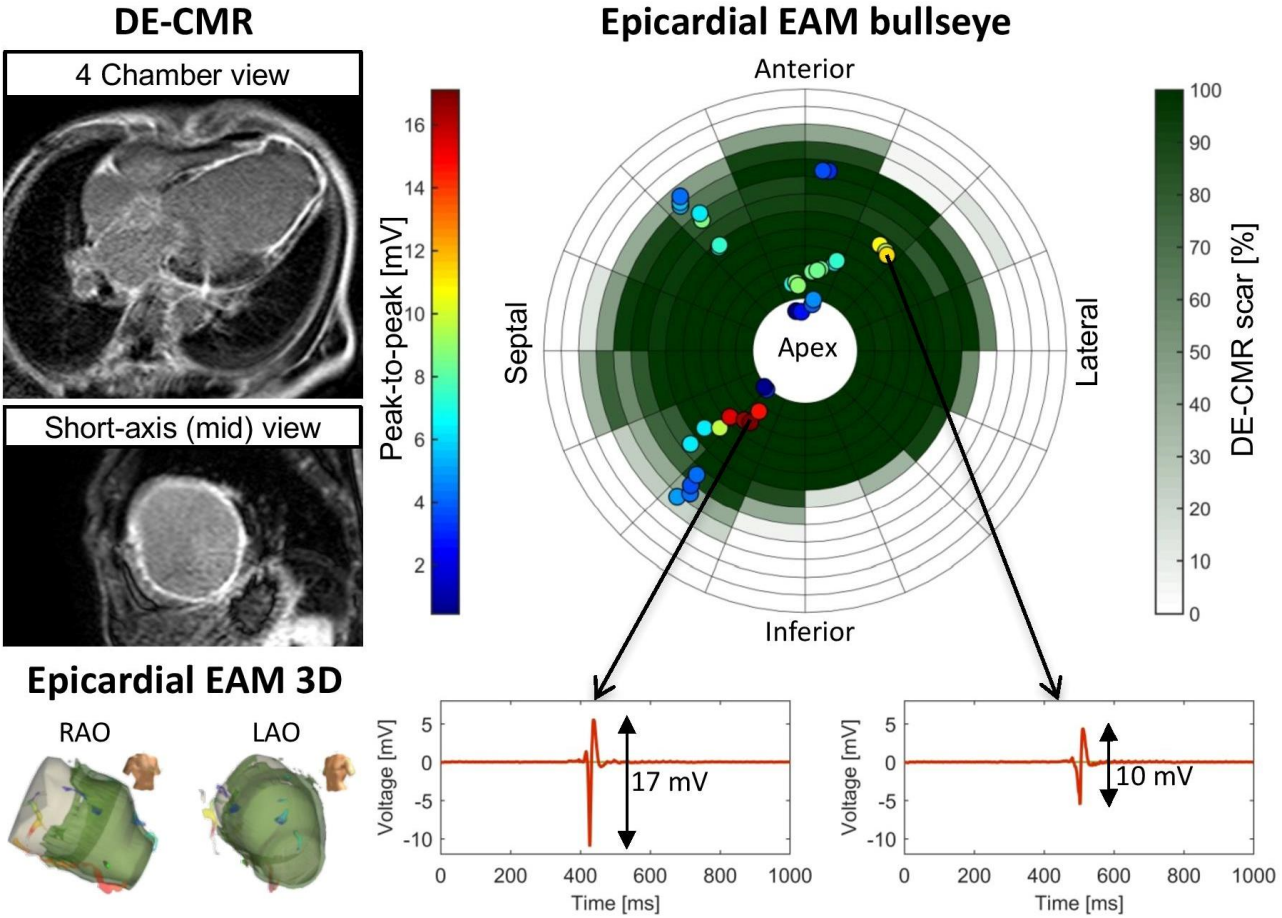
Representative patient (epicardial no.19) with extensive scar on delayed enhancement cardiac magnetic resonance imaging (DE-CMR). All unipolar electrograms were in extensive scar, but normal unipolar voltage amplitudes (UnipV) were still present demonstrating a lack of correlation between low UnipV and scar. EAM = electro-anatomic mapping, LAO = left anterior oblique, RAO = right anterior oblique.

The correlation between UnipV and DE-CMR scar extent was moderate for endocardial (R = -0.33, *p*<0.001) and weak for epicardial measurements (R = -0.07, *p*<0.001). Examples of the lack of correlation between UnipV and scar in patients are represented in Figs 4 and 5.

### Diagnostic accuracy of voltage amplitudes in scar detection

The diagnostic performance of UnipV in identifying any-scar (1–100% scar extent) and segments with >25%, >50%, and >75% scar extent in the overall population is shown in Table 3 and Fig 2B. UnipV predicted the presence of any-scar moderately for the endocardium (AUC = 0.68) and poor for the epicardium (AUC = 0.56). Optimal UnipV thresholds in discriminating any-scar from non-scar only yielded moderate sensitivity and specificity: 8.3 mV (sensitivity 60%, specificity 67%) for endocardial and 7.6 mV (sensitivity 57%, specificity 52%) for epicardial measurements. Endocardial UnipV were moderate predictors for segments with >25% (AUC = 0.72) and >50% (AUC = 0.73) scar extent and fair predictors for segments with >75% scar (AUC = 0.76), while epicardial UnipV were poor predictors for all severities of scar (AUC range: 0.47–0.52).

**Table 3:** Unipolar voltage amplitudes in detecting DE-CMR defined scar.

Substrate	Endocardial UnipV (Group 1)	*p*-value^a^	Epicardial UnipV (Group 2)	*p*-value ^a^
	AUC (CI)		AUC (CI)	
Any-scar				
1–100% scar	0.68 (0.67–0.70)	<0.001	0.56 (0.53–0.59)	<0.001
Scar extent higher than				
>25%	0.72 (0.71–0.74)	<0.001	0.52 (0.48–0.55)	0.424
>50%	0.73 (0.72–0.75)	<0.001	0.54(0.50–0.58)	0.107
>75%	0.76 (0.74–0.78)	<0.001	0.47 (0.40–0.53)	0.399

^a^*P*-values are based on the non-parametric assumption (H0: true area = 0.50). AUC = Area under the curve, CI = confidence interval, UnipV = unipolar voltage.

The diagnostic performance of endocardial UnipV in only detecting >75% scar was additionally evaluated in individual patients. Sixteen out of 33 (48%) endocardial mapped patients had electrograms recorded both in >75% scar and in ≤75% scar and were further analyzed, yielding a mean AUC of 0.66±0.19 (AUC range: 0.23–0.96). From these patients, 6/16 (38%) had an AUC <0.60, indicating poor performance.

## Discussion

In the present study UnipV from either endocardial measurements or epicardial measurements were compared with DE-CMR defined scar in two large cohorts of heterogeneous HF patients, clinically representative for the patients undergoing EAM investigations. The main findings of this study are as follow: (1) UnipV in any-scar are moderately lower than in non-scar, but large overlap exists, particularly for epicardial measurements; (2) UnipV in any-scar were not significantly lower than non-scar in the majority of patients; (3) A large inter-patient variability in non-scar UnipV exists; (4) UnipV are inversely correlated with scar extent, but this was only moderate for endocardial and poor for epicardial measurements; and finally (5) only scar segments with >75% can be properly identified (AUC = 0.76) by endocardial UnipV.

### Voltage amplitudes and scar in pre-clinical studies

Our results show a large overlap between UnipV from non-scar and any-scar segments. Moderate concordance between low UnipV and scar on DE-CMR has also been previously reported. In a recent study, infarct size as defined by endocardial UnipV of <5 mV was compared with scar on DE-CMR in 60 infarcted porcine hearts. [2] Despite the use of a large number of study subjects where complete myocardial scars were employed under controllable circumstances, only a moderate correlation (R = 0.504) between UnipV and scar extent was reported.

The overlap in UnipV from non-scar and scar found in our study was larger in epicardial measurements compared to endocardial measurements, and particularly apparent in segments with less than 75% scar, supposedly areas with non-transmural scar. This indicates that UnipV from epicardial measurements are particularly poor at defining scar, and only segments with extensive infarction (transmural) can be detected properly by endocardial measurements. The observation that epicardial measurements are inferior at predicting scar than endocardial measurements is not entirely new. Lower concordance between bipolar voltage amplitudes and DE-CMR defined scar in epicardial measurements (7/28 maps) compared to endocardial measurements (3/28 maps) has also been reported by Arenal et al. in 31 post-infarct porcine hearts.[14] Additionally Tung et al. demonstrated in 8 post-infarct porcine hearts that epicardial bipolar voltage amplitudes in histological confirmed scar have similar values in fat, but longer electrogram duration, indicating that fat tissue alone can already result in low UnipV values. Epicardial fat was predominantly found in the basal portion of the interventricular course of the left anterior descending artery up to the left atrial appendage and the atria-ventricular groove from the basal portion of the posterior interventricular septum to the basal lateral wall.[6] The coronary venous tributaries we derived our epicardial electrograms from, are typically located in correspondence of these anatomical sites. Therefore, the poorer performance of epicardial electrograms compared to endocardial may be at least partially explained by the presence of epicardial fat.

Taken collectively, pre-clinical data show a high variability in relation between voltage amplitudes and scar, despite that they were performed under controllable circumstances and employed complete infarctions. So even in these controlled conditions the relation between low voltage amplitude and myocardial scar is not very clear, which makes the findings in our study in heterogeneous and clinical HF patients with more complicated scar architecture understandable.

### Voltage amplitudes and scar in clinical studies

In the present study non-scar UnipV both from endocardial as well as epicardial measurements varied considerably between individuals, even in patients without any scar. In addition, UnipV in any-scar were not significantly lower compared to non-scar in the majority of patient, demonstrating again that the use of universal voltage thresholds to detect scar may not be applicable for a substantial number of patients. Previous clinical studies that investigated the relation between voltage amplitudes and scar mostly grouped the data, whereby inter-individual differences remained unnoticed. A few studies employed endocardial mapping to investigate the relation between voltage amplitudes and DE-CMR scar. Edocardial UnipV were compared with scar on DE-CMR in 15 CRT candidates with ischemic cardiomyopathy.[15] UnipV thresholds could differentiate scar from non-scar with a good AUC. However, DE-CMR scar was determined using visual analyses and the total number of DE-CMR segments from subendocardial (*n* = 49) and transmural scar (*n* = 15) was small in comparison with segments from non-scar (*n* = 211), which may have led to less reliable estimations. In a study from Condreanu et al., endocardial UnipV were significantly lower in areas of DE-CMR defined scar compared to non-scar in 10 candidates for VT ablation, but similar to our findings no strong linear correlation between UnipV and scar extent was present.[9] Additionally in another study from Wijnmalen et al., in 15 candidates for VT ablation, endocardial UnipV decreased significantly with increasing scar transmurality, but a high overlap between non-scar and sub-scar groups (with different scar severity) was present. Voltage thresholds were only able to delineate segments with >75% scar properly.[3] In this context, our finding that endocardial UnipV only detected segments >75% scar properly, seems reasonable.

In a study from Piers et al. epicardial UnipV were compared to DE-CMR defined scar and CT defined fat in 10 non-ischemic VT patients. UnipV detected scar properly in areas with <2.8 mm fat, but not in areas with ≥2.8 mm fat.[10] These data support the importance of epicardial fat in modifying voltage amplitudes and may well explain the lack of correlation between epicardial UnipV and scar observed by us.

Taken together, results from previous clinical studies in VT and HF patients confirm the poor correlation between UnipV and scar extent and demonstrate that UnipV may only work for the detecting segments with high scar transmurality. The similarities in results between our cohort and the previous studies are important, because most of the previous studies investigated VT patients, who may have less severe HF than our population. Therefore, the poor correlation between scar and UnipV appears a general property, not limited to a specific patient population.

### Factors affecting voltage amplitudes

The imperfect relation between low voltage amplitude and scar may be understood by multiple factors influencing the electrogram including inter-individual differences, electrode size, catheter orientation, fiber orientation, contact force, fat tissue, LV wall thickness, and activation wavefront.[10, 16–18] Obviously, the electrode size within each study (endocardial or epicardial EAM) was kept constant for all locations. Epicardial UnipV were generally lower compared to the endocardial measurements, possibly because of difference in EAM systems, electrode size or shape, and the presence of epicardial fat. The large inter-patient differences can however only be explained by differences in tissue properties, because it is hard to imagine that contact force is consistently lower in one patient than another. Site-to site variability in contact force and electrode orientation may furthermore explain intra-patient differences. Fiber orientation and activation wavefront can cause differences in voltage amplitudes, since these differences may occur locally and between patients. The role of epicardial fat seems supported by the weaker correlation between UnipV and scar in the epicardial measurements compared to the endocardial measurements. Besides epicardial fat, other possible mechanisms might have contributed to this observation. First, a typical non-transmural star is often located at the endocardium, and it is likely that epicardial measurements are less influenced by endocardial scar due to the interfering myocardium, than endocardial measurements. However, the larger field of view for unipolar recordings compared to bipolar recordings should (at least partially) compensate for this effect.[19] Second, epicardial measurements were limited to the anatomy of the coronary veins, during which the catheter was mostly oriented parallel to the long axis of the heart along the anatomy of the coronary tributaries and are therefore more prone to poor contact force, due to limited freedom of catheter movement compared to endocardial measurements. However, all the above elaborated factors are unlikely to affect endocardial measurements, and cannot fully explain the moderate correlation between endocardial UnipV and scar.

### Clinical implications

To date, the majority of EAM vendors incorporate the option to set voltage thresholds for scar delineation. Yet, evidence that these thresholds delineate scar accurately is scarce. In our large population, normal median UnipV already ranged tremendously, which argues against the use of universal voltage thresholds. This idea is supported by the large inter-individual variation in bipolar voltage amplitudes reported by Cassidy et al.[17] In case of regional scar, regional differences in amplitudes may be helpful, but only to predict (almost) transmural scars. Therefore, associating low voltage amplitudes with scar should be done with caution and may only work in tissue with extensive scarring. Cautiousness is also in place in the detection of arrhythmogenic substrate and critical sites of VT ablation, as these procedures also could rely on low voltage areas.

### Limitations

In our DE-CMR analyses approach, a segment was categorized as any-scar when 1–100% delayed enhancement was present. While this approach may have led to smaller UnipV differences between non-scar and any-scar, the weak correlation found between UnipV and scar extent is not affected by thresholds used for scar detection. Additionally, sub-analyses for scar groups with different severities of scar were carried out.

EAMs were acquired with a NOGA catheter for the endocardial measurements and a guidewire in the coronary veins for the epicardial measurements. There are other techniques to perform EAM, but the fact that two different techniques show similar results suggests that the present results can be largely extrapolated to other EAM systems. However, UnipV thresholds and accuracy values obtained in the present study need to be tested and validated in an independent population, using other EAM systems, albeit the measured UnipV should not depend on the mapping system utilized.

Other electrogram characteristics such as fractionation and long duration could also play a role in scar detection. Whether these parameters would be superior in identifying scar than UnipV is left to be investigated.

## Conclusion

UnipV in scar defined by DE-CMR are moderately lower than in non-scar, but large overlap in UnipV between non-scar and scar and high inter-patient variability exists, particularly in patients undergoing epicardial measurements and segments with lower scar extent. The only reasonably scar assessment can be expected when using endocardial UnipV measurements for the detection of segments with >75% scar. Based on the current results, the use of low UnipV for identification of myocardial scar should be done with caution.
